# Leptospirosis in rats and livestock in Bantul and Gunungkidul district, Yogyakarta, Indonesia

**DOI:** 10.14202/vetworld.2022.1449-1455

**Published:** 2022-06-12

**Authors:** Sunaryo Sunaryo, Dwi Priyanto

**Affiliations:** 1 National Research and Innovation Agency of Indonesia, Jakarta, Indonesia; 2 Banjarnegara Health Research and Development Unit, National Institute of Health Research and Development, Indonesian Ministry of Health, Jakarta, Indonesia

**Keywords:** cattle, leptospirosis, prevalence, rats

## Abstract

**Background and Aim:**

The animal reservoir of leptospirosis is comprised of both domestic and wild mammals, with rats known as the most important in the spread of the disease. The occurrence of this reservoir in residential areas increases the potential for leptospirosis transmission. This study aimed to investigate the type of reservoirs and estimate the prevalence of leptospirosis in rats and livestock animals in Bantul and Gunungkidul districts, Special Region of Yogyakarta Province, Indonesia.

**Materials and Methods:**

This research utilized a cross-sectional study design. Rat trapping and livestock (cattle, goat, and sheep) blood surveys were conducted at four locations in each district. Samples of rat renal and livestock blood serum were examined using the polymerase chain reaction technique to determine the presence of *Leptospira* bacteria. The data were analyzed descriptively by describing the species of rats trapped, the types of cattle, and the prevalence of *Leptospira* in the sample.

**Results:**

The rat species infected with *Leptospira* in Bantul district consisted of *Rattus tanezumi* 4.8% (3/63); *Rattus norvegicus* 12.5% (2/16); *Bandicota indica* 28.6% (2/7); and *Bandicota bengalensis* 50.0% (1/2). No rats were found to be positive for *Leptospira* in Gunungkidul district. The prevalence of *Leptospira* in cattle was 63.64% (7/11) in Bantul district and 50.00% (8/16) in Gunungkidul district. In goats and sheep, the prevalence of *Leptospira* was 22.22% (2/9) in Bantul district and 45.16% (14/31) in Gunungkidul district.

**Conclusion:**

The potential exists for transmission of leptospirosis from rats and cattle in Bantul and Gunungkidul Districts. It is necessary to increase leptospirosis awareness. Community education, especially for livestock farmers, needs to be improved to prevent the transmission of leptospirosis from livestock.

## Introduction

Leptospirosis is a re-emerging disease caused by pathogenic bacteria of the genus *Leptospira*. This zoonosis has the most widespread worldwide distribution, with more than 1 million cases experiencing severe disease and approximately 60,000 deaths reported annually [1]. Leptospirosis is classified as a neglected disease [2], with wide distribution and cases reported in both urban [3] and rural areas [4]. Leptospira reservoirs in both types of settlements are responsible for the high prevalence rates.

*Leptospira* spp. infection occurs through two routes: The direct route of contact with urine or tissue from infected animals and the indirect route through an environment (usually moist) contaminated with the urine of infected animals [5, 6]. Domestic animals play an important role as hosts or reservoirs of the bacteria [7]. *Leptospira* can be found in various domestic mammals including rodents [8, 9], cattle [7], sheep [10], dogs [11], cats [12], and other livestock [13]. Interaction between humans and these animals is a risk factor for transmission.

Rodents are known to be the main reservoir of leptospirosis [9, 14]. This group consists of many species, some of which are domesticated, so they have a high potential to transmit leptospirosis to humans [15]. *Rattus tanezumi* is the most dominant species found in residential areas [16]. They can occupy homes by nesting on roofs, thus allowing them to escape eradication attempts. *Rattus norvegicus*, better known as the “sewer rat” in Indonesia, is a species that is commonly found in residential areas with poor sanitation [17]. Several studies report that this species is a reservoir of leptospirosis in urban areas of Indonesia [18–20]. *Mus musculus* [19], *Rattus argentiventer* [21], and *Bandicota indica* [22] are also abundant species, especially in tropical agricultural areas.

The Special Region of Yogyakarta Province has reported incidents of leptospirosis in the previous 5 years [23]. Two districts: Gunungkidul and Bantul continue to be recognized for endemic leptospirosis. The number of leptospirosis cases in Gunungkidul district is lower than in Bantul, but the mortality rate is higher, exceeding 20%. Indeed, it was 100% (4/4) in 2016. Over the past 5 years, the leptospirosis incidence rates in Bantul district have ranged from 80 to 100 cases. However, the mortality rate has been reduced to below 6%. As a postscript, the Bantul District Health Office reported a leptospirosis outbreak in 2011 [24]. The risk factors for leptospirosis in Bantul and Gunungkidul districts include exposure to the various leptospirosis reservoirs found in the community.

Leptospirosis is still a neglected disease in Indonesia, particularly in Yogyakarta. The knowledge levels of people who are at risk of being exposed to leptospirosis, and local health workers, need to increase. Evidence showing that the risk of exposure to leptospirosis exists in the environment is expected to raise this awareness. This study aimed to estimate the prevalence of leptospirosis in many types of reservoirs, especially domestic animals such as rats and livestock.

## Materials and Methods

### Ethical approval

The study was approved by Indonesian Health Research Ethics Committee, National Institute of Health Research and Development (number LB.02.01/2/KE.090/2019). The National and Political Unitary Agency of Yogyakarta gave the consent and permission to conduct the study.

### Study period and location

**Figure-1:**
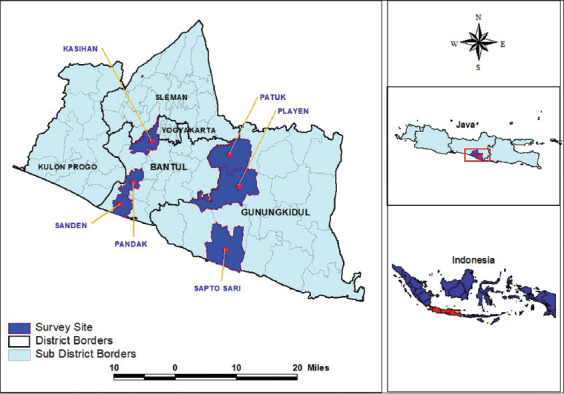
Survey site map [Source: Ina Geospatial Maps 2018].

A cross-sectional study was conducted from January to November 2019 in Bantul and Gunungkidul districts, Special Region of Yogyakarta Province. The survey was conducted in four subdistricts (Figure-1). Purposive random sampling was applied to select a survey site based on the Local Health Office’s leptospirosis case record.

### Animals and sampling

Rats were captured in settlement areas, including household areas, rice fields, and gardens around houses. Rat trapping was performed for four consecutive nights at each survey site. Species identification, blood, and renal samples were taken directly at the on-site locations. The captured rats were immediately identified and then dissected; the kidneys were put into tubes containing phosphate-buffered saline and stored at 4°C. Livestock animals had blood drawn through the jugular vein. The blood was immediately placed into tubes containing ethylenediaminetetraacetic acid and stored at 4°C.

### *Leptospira* detection

Examination for the presence of leptospires was carried out in the Laboratory of Banjarnegara Health Research and Development Unit using the polymerase chain reaction (PCR) method with the Lipl32 gene target, run according to the protocols published by Levett *et al*. [25] and Vedhagiri *et al*. [26].

### Statistical analysis

Data were analyzed descriptively by the proportion of rats and livestock species that were obtained during the study period, and the prevalence of leptospirosis in the samples examined. Rat density data at the study sites were determined by the trap success rates, calculated by the total number of rats caught divided by the number of traps and the number of capture days.

## Results

**Figure-2:**
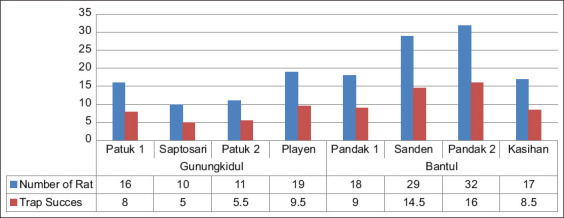
Number of rats captured and trap success indices of each survey site in Gunungkidul and Bantul district.

Fifty-six rats were captured from four survey sites in Gunungkidul district, that is, Patuk 1 (n = 16); Saptosari (n = 10); Patuk 2 (n = 11); and Playen (n = 19). The relative density of rats described by the rate of successful traps in this district was 5-9.5%. Trapping in Bantul district captured more rats from Pandak 1 (n = 18), Sanden (n = 29), Pandak 2 (n = 32), and Kasihan (n = 17), which is indicated by its successful trap rate (Figure-2).

**Figure-3:**
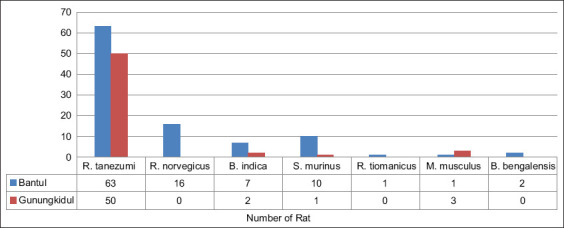
The species of rats captured from survey sites in Gunungkidul and Bantul district.

The rats captured at four locations spread across each district of Gunungkidul and Bantul were identified as six species, that is, *R. tanezumi*, *R. norvegicus*, *B. indica*, *M. musculus*, *Bandicota bengalensis*, and *Rattus tiomanicus*. This survey also obtained one non-rodent species, the shrew *Suncus murinus*, which is also known to have the potential to be infected with *Leptospira*. *R. tanezumi* was the most abundant and dominant species caught in Gunungkidul and Bantul districts (Figure-3).

### *Leptospira* examination from rat renal samples

The results of the examination of *Leptospira* positive rat renal samples based on species and their sampling site by PCR are described in Table-1.

**Table 1:** *Leptospira* examination by PCR methods of rats samples from Bantul district.

Species	n	Positive	Percentage	Survey site
*Rattus tanezumi*	63	3	4.8	Kasihan and Sanden
*Rattus norvegicus*	16	2	12.5	Kasihan and Sanden
*Bandicota indica*	7	2	28.6	Kasihan and Pandak
*Suncus murinus*	10	0	0	-
*Rattus tiomanicus*	1	0	0	-
*Mus musculus*	1	0	0	-
*Bandicota bengalensis*	2	1	50.0	Sanden

PCR=Polymerase chain reaction

Rats caught in Bantul district infected with *Leptospira* consisted of four species: *B. indica* (28.6%: 2/7), *R. norvegicus* (12.5%: 2/16), *R. tanezumi* (4.8%: 3/63), and *B. bengalensis* (50.0%: 1/2). *Leptospira*-positive rats were trapped at three locations: Kasihan, Sanden, and Pandak 2. In addition, *Leptospira* detection in rats from Gunungkidul district showed negative results in all samples.

### *Leptospira* examination from livestock blood samples

The examination of 67 samples of livestock blood found 31 samples (46.27%) to be positive for *Leptospira*. These samples were obtained from 15 cattle and 16 goats/sheep (Tables-2 and 3).

**Table 2:** *Leptospira* examination by PCR methods of livestock blood samples from Bantul district.

Livestock	n	Positive	Percentage	Survey site
Cow	11	7	63.64	Pandak
Goat/Sheep	9	2	22.22	

PCR=Polymerase chain reaction

**Table 3:** *Leptospira* examination by PCR methods of livestock blood samples from Gunungkidul district.

Livestock	n	Positive	Percentage	Survey site
Cow	16	8	50.00	Saptosari and Playen
Goat/Sheep	31	14	45.16	

PCR=Polymerase chain reaction

## Discussion

The density of rats is directly proportional to the risk of *Leptospira* transmission. In this study, the density of rats was described in terms of the relatively successful trap capture rates. A trapping location was classified as having a high density of rats if the trap success rate was above 7%. The average trap success in Gunungkidul was 7% (5-9.5%), while in Bantul district, it was 12.25% (8.5-16%). Regardless of the bias caused by differences in the number of catchers and the equipment used, both districts have high rat densities, even though the rat capture rate in Gunungkidul was lower than in Bantul district. In Gunungkidul district, the study was conducted in a low-density housing area, while in Bantul district, it was conducted in a densely populated location. Dense settlements provide more food sources, such as waste and leftover food for rats, especially domestic rats such as *R. norvegicus* and *R. tanezumi*.

The genus *Rattus* seems to benefit from urbanization and thrive in an urban and peridomestic environment. *R. tanezumi* was the most frequently caught species across all study sites. This is reasonable, considering that the survey was conducted at residential locations. *R. tanezumi* is known as a “house rat” which has a wide range of habitats and nests in attics and other hidden places, both inside and outside houses. These species can climb house walls, so can easily go in and out for many purposes, such as foraging, mating, and nesting. The second most captured species was *R. norvegicus*, which is a “sewer rat” and is widely found in urban environments. This species does not have the ability to climb, so its access in and out of houses is relatively limited compared with *R. tanezumi*. However, in urban areas, mostly in developing countries, this species was generally found in slums and in areas with poor sanitation, such as markets, shopping complexes, and surrounding areas. In this study, *R. norvegicus* was captured in survey sites in both Bantul and Gunungkidul. In relation to zoonosis, *R. norvegicus* has been reported to act as a major reservoir for leptospirosis [9].

*B. indica* is commonly found in agricultural areas or in settlements adjacent to agricultural areas (e.g., rice and farm fields). This species has also been reported to transmit leptospirosis. The results of the trapping activities of this study found only two *B. indica* in Gunungkidul district and seven in Bantul district. The survey site in Bantul was a settlement area, with many rice and farm fields surrounding the residents’ houses. This is the common habitat of *B. indica*. It is noteworthy that the rice and farm fields in Bantul are narrower than those in Gunungkidul district, which causes the habitat range of *B. indica* to increasingly narrow. This results in the activity of this species being closer to residential areas. *B. indica* was mostly caught in Bantul district, probably due to the narrowing of its habitat range, making it easier to capture.

Other species captured were *R. tiomanicus*, *M. musculus*, and *B. bengalensis*. These three species were caught in settlement areas in Bantul district. This finding was in contrast with the study of “Rikhus Vektora” in South Sumatra, which found the species in areas far from settlements [16]. *R. tiomanicus* is a type of tree rat and its natural habitat is outside of houses, and is commonly found in plantation areas or rice fields adjacent to rivers, while *B. bengalensis* is a species that occupies garden or rice field habitats that are identical to rural areas. Data show that these two species were caught in Bantul district, the possible explanation being the same as in *B. indica* phenomenon, which was also caught more frequently in Bantul district. *M. musculus* is a more cosmopolitan species, but many studies report that this species is becoming increasingly difficult to find in residential areas, especially in Java. This may be related to competition from other species, especially *R. tanezumi*, resulting in a decreasing population.

The prevalence of *Leptospira* in rats is influenced by the rate of transmission between rats, both within and between species. In areas of high-level rat infestation, the potential for *Leptospira* transmission increases with more frequent interactions between rats. The trap success rate data showed that the rat infestation in Gunungkidul district was lower than in Bantul district. This low rat population resulted in a lower rate of leptospirosis transmission between them. In addition, none of the rats caught in Gunungkidul were infected with *Leptospira*.

In this study, all the positive rats for Leptospira were caught in Bantul district, and these rats occupied denser settlements than those in Gunungkidul district. The relatively large number of houses per capture area and the lower areas of rice and farm fields, which are their preferred habitats, make interactions between rats more frequent and ultimately increases the potential for *Leptospira* transmission between them.

Rats are maintenance hosts of leptospires, and these animals act as reservoirs for *Leptospira* bacteria over relatively long periods of time. This is enabled by the fact that rats do not show clinical signs even though they are infected with *Leptospira*. *Leptospira* bacteria are deposited in the renal tubules and excreted in the urine of rats. Thus, the presence of infected rats has the potential to transmit the pathogen to other rats and animals and therefore has the potential to be transmitted to humans. The species of rats infected with *Leptospira* in Bantul district were *R. tanezumi* (4.8%: 3/63), *R. norvegicus* (12.5%: 2/16), *B. indica* (28.6%: 2/7), and *B. bengalensis* (50%: 1/2). These results were consistent with several studies conducted in other areas. *R. tanezumi*, which is the species with the highest density in settlement locations, has also been confirmed as a *Leptospira* reservoir in Banyumas, with a prevalence of 2.7% [27], Minahasa 9.0% [8], Pati 40% [28], and Semarang city 8.3% [17].

The prevalence of leptospirosis in this study was relatively low, and considering the small sample size, high confidence intervals could not be determined. However, this has also been the case in similar studies in other areas which encountered the same problem. Despite these limitations, the figures from these various studies can still be compared equally.

*R. norvegicus* was caught in fewer numbers than *R. tanezumi*, but the prevalence of *Leptospira* in this species was higher. This finding correlates with data from several other studies. The prevalence of *R. norvegicus* infected with leptospirosis was 86.7% in Semarang [17]; 79% in Salvador, Brazil, 12% in Vancouver, Canada [29]; and 25% in Vienna, Austria [30]. The prevalence of *Leptospira* in *B. indica* in this study was the highest, in line with a study in Bangladesh which reported that this species was the most prevalent at 66.7% [31]. *B. bengalensis* had the highest prevalence of *Leptospira*, with one positive out of two samples examined (50%). This species was caught in small numbers, so the prevalence rate was high. As a caution, it is important to be aware that a small population in a relatively narrow area affects the interspecies transmission of *Leptospira*, considering that rats interact with other rats not only in terms of competing for food or habitat dominance but also in terms of breeding. The small number of individuals of one species in a narrow habitat makes this interspecies interaction more intense, resulting in an increased potential for transmission. Another study reported that *B. bengalensis* was a leptospirosis reservoir in Bangladesh (31.4%) [31], Sri Lanka (29%) [32], and India (37.2%) [33].

*S. murinus* is not a member of the Rodentia group, but is a shrew and an insectivore. However, several studies have reported that this species also has the potential to transmit leptospirosis, so its existence in the environment needs to be monitored. In this study, PCR detection showed that none of *S. murinus* were infected with leptospirosis. However, *S. murinus* was reported to be infected with a prevalence of 6.7% with leptospirosis serovar Cynopteri in Banyumas district [27]. In addition, a greater prevalence (25%) was reported by a study conducted in Malaysia [34].

The livestock samples confirmed in this study included cattle, goats, and sheep. The data showed that the prevalence of *Leptospira* in livestock in Bantul and Gunungkidul district was high, with the prevalence of *Leptospira* in cattle in Bantul district at 63.64%, while in goats and sheep, the prevalence was 22.22%. This corresponds with the findings in Gunungkidul district where the prevalence in cattle was 50.00% and 45.16% in goats and sheep. The potential for transmission of leptospirosis from cattle to humans, especially stocks farmers, needs particular consideration. Importantly, the study results in Kulonprogo district revealed that most stock farmers still do not have adequate knowledge about *Leptospira* [23].

Similar to transmission to humans, *Leptospira* infection in animals occurs due to direct contact with urine or indirect contact with a contaminated environment. The high mobility conferred by leptospiral periplasmic flagella allows the bacteria to invade the host organism by active penetration through injured skin or intact mucosa. When colonizing the kidneys, leptospires are excreted in urine and may then contaminate soil, surface water, puddles, streams, and rivers [35]. The study location was a suburban area with poor environmental sanitation. This type of environment allows for the transmission of leptospirosis among various types of reservoirs, including rats, domestic animals, and humans.

*Leptospira* infection in cattle is reported to cause disease with mild-to-severe symptoms. A survey conducted in Kulonprogo district from 2011 to 2013 found that out of all of the corrals investigated, 5.6% had at least one individual animal infected with *Leptospira* among their livestock. The prevalence of cattle infected with *Leptospira* was 3.7%, while that of small ruminants was 3.3%. The most dominant serovars found were Hardjo and Icterohemorrhagie [23]. Kulonprogo district is another district in Yogyakarta Province and has similarities in geography, environment, and the techniques used by farmers in managing livestock, which makes it similar to Bantul and Gunungkidul.

Several studies have shown similar results to this study. A study conducted on sheep in Brazil reported that 24.74% were infected with *Leptospira*, with Hardjo being the dominant serovar and all positive samples resulting from infection with the species *Leptospira interrogans* [10]. Studies conducted in Malaysia reported that the leptospires prevalence in cattle was 11.75%, in goats was 11.2%, and in sheep was 5.03%. The reported serovars were Hardjo, Hebdomadis, and Pomona [7]. Research in Sri Lanka reported that 11% of female rats and 8% of male rats were infected with *Leptospira*, 9% of the cattle tested were confirmed *Leptospira* positive, and the prevalence in buffaloes was 20% [36]. Another study in Malaysia using a serological examination method found a very high prevalence of leptospires in cattle (81.7%), with Sarawak being the most dominant serovar [37]. This study indicated that the incidence of leptospirosis in cattle was very high, although it does not cause death in cattle. A study conducted in Kelantan, Malaysia, reported that the prevalence of leptospires in cattle was 14.16%; in goats was 11.20%; and in sheep was 5.03% [7].

All samples were analyzed using the PCR method with the *Lipl32* gene targeted. This is a specific gene for pathogenic leptospires, so the positive samples detected were likely due to pathogenic *Leptospira* infection. However, a weakness of this study is that no serovar data were obtained, making it difficult to compare with the results of other studies or to compare them with *Leptospira* serovars known to infect humans at the study sites. In general, it can be concluded that there is the potential for transmission of leptospirosis from rats and livestock at the study sites, referring to the results that pathogenic leptospires were indeed proven to be present in these domestic animals.

All the rats caught in Gunungkidul district showed negative results for *Leptospira*, in contrast with the positive results of the livestock. These data prove that action is needed to prevent transmission among farm communities. Leptospirosis in cattle can cause economic losses to farmers and the risk of transmitting leptospirosis to the farmers themselves. Leptospirosis in cattle has been reported to cause disturbances in the female estrous cycle [38] and failure of fetal development [35]. Likewise, in goats and sheep, leptospirosis causes reproductive disorders, including fetal abortion and decreased milk production [39]. Investigation in Bantul district revealed *Leptospira* prevalence in both rats and cattle. The potential for leptospirosis transmission from domestic and wild animals was higher in Bantul district than in Gunungkidul. This result is directly proportional to the cases of leptospirosis seen in humans. Each year Bantul district reports relatively higher case numbers than Gunungkidul district.

## Conclusion

This study has not been able to explain how closely related leptospirosis is in rats and livestock quantitatively nor the similarity of *Leptospira* serovars in the research subject animal groups. Despite this, the potential exists for transmission of leptospirosis from rats and cattle in Bantul and Gunungkidul districts. Rats that were confirmed positive for pathogenic *Leptospira* in Bantul district were *R. tanezumi*, *R. norvegicus*, *B. indica*, and *B. bengalensis*. In addition, the investigated cattle, goats, and sheep proved to be potential reservoirs for leptospirosis in both districts.

It is necessary to increase leptospirosis awareness by increasing the quality and quantity of leptospirosis surveillance, especially surveillance based on reservoir animals. Community education, especially for stock farmers, needs to be improved to prevent the transmission of leptospirosis from livestock.

## Authors’ Contributions

SS: Supervised the study and drafted the manuscript. DP: Carried out sample collections and laboratory analysis, data collection and analysis, and made critical comments on the manuscript. All authors have read and approved the final manuscript.

## References

[1] Narkkul U, Thaipadungpanit J, Srisawat N, Rudge J.W, Thongdee M, Pawarana R, Pan-Ngum W (2021). Human, animal, water source interactions and leptospirosis in Thailand. Sci. Rep.

[2] Karpagam K.B, Ganesh B (2020). Leptospirosis:A neglected tropical zoonotic infection of public health importance an updated review. Eur. J. Clin. Microbiol. Infect. Dis.

[3] Blasdell K.R, Morand S, Perera D, Firth C (2019). Association of rodent-borne *Leptospira* spp. with urban environments in Malaysian Borneo. PLoS Negl. Trop. Dis.

[4] Suut L, Mazlan M.N.A, Arif M.T, Yusoff H, Abdul Rahim N.A, Safii R, Suhaili M.R (2016). Serological prevalence of leptospirosis among rural communities in the Rejang Basin, Sarawak, Malaysia. Asia Pac. J. Public Health.

[5] Levett P.N (2019). Leptospirosis. Clin. Microbiol. Rev.

[6] Mwachui M.A, Crump L, Hartskeerl R, Zinsstag J, Hattendorf J (2015). Environmental and behavioural determinants of leptospirosis transmission:A systematic review. PLoS Negl. Trop. Dis.

[7] Abdul Rahman M.S, Bejo S.K, Zakaria Z, Hassan L, Roslan M.A (2020). Seroprevalence and distribution of leptospiral serovars in livestock (cattle, goats, and sheep) in flood-prone Kelantan, Malaysia. J. Vet. Res.

[8] Lobo L.T, Koraag M.E, Widjaja J, Joharina A.S, Pratiwi A.P (2020). Leptospirosis on rats in Minahasa district, North Sulawesi 2016. J. Vektor Penyakit.

[9] Boey K, Shiokawa K, Rajeev S (2019). *Leptospira* infection in rats:A literature review of global prevalence and distribution. PLoS Negl. Trop. Dis.

[10] Almeida D.S, Paz L.N, De Oliveira D.S, Silva D.N, Ristow P, Hamond C, Costa F, Portela R.W, Estrela-Lima A, Pinna M.H (2019). Investigation of chronic infection by *Leptospira* spp. In asymptomatic sheep slaughtered in a slaughterhouse. PLoS One.

[11] Lau S, Low K, Khor K, Roslan M, Bejo S, Radzi R, Bahaman A (2016). Prevalence of leptospirosis in healthy dogs and dogs with kidney disease in Klang Valley, Malaysia. Trop. Biomed.

[12] Murillo A, Goris M, Ahmed A, Cuenca R, Pastor J (2020). Leptospirosis in cats:Current literature review to guide diagnosis and management. J. Feline Med. Surg.

[13] Zakharova O.I, Korennoy F.I, Toropova N.N, Burova O.A, Blokhin A.A (2020). Environmental risk of leptospirosis in animals:The case of the Republic of Sakha (Yakutia), Russian Federation. Pathogens.

[14] Nova R.I.T, Susanna D, Warsito G.M (2020). The presence of rodents infected with *Leptospira* bacteria in various countries and the leptospirosis potential in humans:A systematic review. Malays. J. Public Health Med.

[15] Priyanto D, Raharjo J, Rahmawati (2020). Rat domestication:Study on foraging and nesting behavior. Balaba.

[16] Supranelfy Y, S N.H, Oktarina R (2019). Analysis of environmental factors on distribution of rats which confirmed as a reservoir in three districts in South Sumatera Province. Vektora J. Vektor Reserv. Penyakit.

[17] Sholichah Z, Ikawati B, Marbawati D, Khoeri M.M, Ningsih D.P (2021). The role of *Rattus norvegicus* from rat and suncus group as the main transmitter of *Leptospira* in Semarang. J. Vektor Penyakit.

[18] Byers K.A, Lee M.J, Patrick D.M, Himsworth C.G (2019). Rats about town:A systematic review of rat movement in urban ecosystems. Front. Ecol. Evol.

[19] Rothenburger J.L, Himsworth C.H, Nemeth N.M, Pearl D.L, Jardine C.M (2017). Environmental factors and zoonotic pathogen ecology in urban exploiter species. Ecohealth.

[20] de Jesus Santos N, Sousa E, Reis M.G, Ko A.I, Costa F (2017). Rat infestation associated with environmental deficiencies in an urban slum community with a high risk of leptospirosis transmission. Cad. Saude Publica.

[21] Sudarmaji, Herawati N.A (2018). Breeding Ecology of the Rice Field Rat (*Rattus argentiventer* Rob and Kloss, 1916) in Irrigated Rice Ecosystem in Indonesia. AIP Conference Proceedings.

[22] Bordes F, Caron A, Blasdell K, de Garine-Wichatitsky M, Morand S (2017). Forecasting potential emergence of zoonotic diseases in South-East Asia:network analysis identifies key rodent hosts. J. Appl. Ecol.

[23] Widiasih D.A, Lindahl J.F, Artama W.T, Sutomo A.H (2021). Leptospirosis in ruminants in Yogyakarta, Indonesia:A serological survey with mixed methods to identify risk factors. Trop. Med. Infect. Dis.

[24] Sunaryo, Ikawati B (2012). Vulnerable leptospirosis mapping model based on risk factor and trap success in Bantul, Yogyakarta. J. Ekol. Kesehat.

[25] Levett P.N, Morey R.E, Galloway R.L, Turner D.E, Steigerwalt A.G, Mayer L.W (2005). Detection of pathogenic leptospires by real-time quantitative PCR. J. Med. Microbiol.

[26] Vedhagiri K, Natarajaseenivasan K, Chellapandi P, Prabhakaran S.G, Selvin J, Sharma S, Vijayachari P (2009). Evolutionary implication of outer membrane lipoprotein-encoding genes ompL1, UpL32 and lipL41 of pathogenic *Leptospira* Species. Genomics Proteomics Bioinforma.

[27] Ramadhani T, Widyastuti D, Priyanto D (2016). Determination of *Leptospira* serovar in reservoir in Banyumas district. J. Ekol. Kesehat.

[28] Sholichah Z, Rahmawati R (2017). Distribution of pathogenic *Leptospira* infection in rats and shrews in flood area of Pati district and endemic area of Boyolali district. Balaba.

[29] Minter A, Himsworth C.G, Byers K.A, Childs J.E, Ko A.I, Costa F (2019). Tails of two cities:Age and wounding are associated with carriage of *Leptospira interrogans* by Norway rats (*Rattus norvegicus*) in ecologically distinct urban environments. Front. Ecol. Evol.

[30] Desvars-Larrive A, Smith S, Munimanda G, Bourhy P, Waigner T, Odom M, Gliga D.S, Walzer C (2020). Prevalence and risk factors of *Leptospira* infection in urban brown rats (*Rattus norvegicus*), Vienna, Austria. Urban Ecosyst.

[31] Krijger I.M, Ahmed A.A.A, Goris M.G.A, Koerkamp P.W.G, Meerburg B.G (2019). Prevalence of *Leptospira* infection in rodents from Bangladesh. Int. J. Environ. Res. Public Health.

[32] Yathramullage S, Meegaskumbura S (2016). *Leptospira* reservoirs among small mammals in Sri Lanka. J. Bacteriol. Mycol.

[33] Kanagavel M, Margreat A.A.P, Arunkumar M, Prabhakaran S.G, Shanmughapriya S, Natarajaseenivasan K (2016). Multilocus sequence typing (MLST) of leptospiral strains isolated from two geographic locations of Tamil Nadu, India. Infect. Genet. Evol.

[34] Azhari N.N, Ramli S.N.A, Joseph N, Philip N, Mustapha N.F, Ishak S.N, Mohd-Taib F.S, Md Nor S, Yusof M.A, Mohd Sah S.A, Mohd Desa M.N. Bin, Bashiru G, Zeppelini C.G, Costa F, Sekawi Z, Neela V.K (2018). Molecular characterization of pathogenic *Leptospira* sp. in small mammals captured from the human leptospirosis suspected areas of Selangor state, Malaysia. Acta Trop.

[35] Loureiro A.P, Lilenbaum W (2020). Genital bovine leptospirosis:A new look for an old disease. Theriogenology.

[36] Denipitiya D.T.H, Chandrasekharan N.V, Abeyewickreme W, Hartskeerl R.A, Hapugoda M.D (2017). Identification of cattle, buffaloes and rodents as reservoir animals of *Leptospira* in the District of Gampaha, Sri Lanka. BMC Res. Notes.

[37] Daud A, Fuzi N.M.H, Arshad M.M, Kamarudin S, Mohammad W.M.Z, Amran F, Ismail N (2018). Leptospirosis seropositivity and its serovars among cattle in Northeastern Malaysia. Vet. World.

[38] Libonati H.A, Santos G.B, Souza G.N, Brandão F.Z, Lilenbaum W (2018). Leptospirosis is strongly associated with estrus repetition on cattle. Trop. Anim. Health Prod.

[39] Ali S, Zhao Z, Zhen G, Kang J.Z, Yi P.Z (2019). Reproductive problems in small ruminants (sheep and goats):A substantial economic loss in the world. Large Anim. Rev.

